# Fast methods for training Gaussian processes on large datasets

**DOI:** 10.1098/rsos.160125

**Published:** 2016-05-11

**Authors:** C. J. Moore, A. J. K. Chua, C. P. L. Berry, J. R. Gair

**Affiliations:** 1 Institute of Astronomy, Madingley Road, Cambridge CB3 0HA, UK; 2 School of Physics and Astronomy, University of Birmingham, Birmingham B15 2TT, UK; 3 School of Mathematics, University of Edinburgh and Biomathematics and Statistics Scotland, James Clerk Maxwell Building, Peter Guthrie Tait Road, Edinburgh EH9 3FD, UK

**Keywords:** Gaussian processes, regression, data analysis, inference

## Abstract

Gaussian process regression (GPR) is a non-parametric Bayesian technique for interpolating or fitting data. The main barrier to further uptake of this powerful tool rests in the computational costs associated with the matrices which arise when dealing with large datasets. Here, we derive some simple results which we have found useful for speeding up the learning stage in the GPR algorithm, and especially for performing Bayesian model comparison between different covariance functions. We apply our techniques to both synthetic and real data and quantify the speed-up relative to using nested sampling to numerically evaluate model evidences.

## 1. Introduction

A wide range of commonly occurring inference problems can be fruitfully tackled using Bayesian methods. A particularly common inference problem is that of regression; determining the relationship of a control variable *x* to an output variable *y* given a set of measurements of {*y*_*i*_} at points {*x*_*i*_}. The solution requires a model *y*=*f*(*x*), which allows us to predict the value of *y* at an untested value of *x*. From a Bayesian standpoint, this can be achieved using Gaussian processes (GPs): a GP is a collection of random variables, of which any finite subset have a joint Gaussian probability distribution [1].

Gaussian process regression (GPR) is a powerful mathematical technique for performing non-parametric regression in a Bayesian framework [1–5]. The key assumption underpinning the method is that the observed dataset being interpolated is a realization of a GP with a particular covariance function. This assumption presents us with a challenge: how do we choose the covariance function which gives the best interpolant?

The process of choosing the covariance function is known as *learning*, or *training* of the GP. In this training process, it is necessary to compute the inverse of the covariance matrix (the matrix formed by evaluating the covariance function pairwise between all *n* observed points). The time taken to evaluate the inverse of the covariance matrix scales as $O(n^{3})$ [1], where *n* is the number of points being interpolated; this has typically restricted the application of GPR to smaller problems ($n≲10^{5}$), although work has been done on extending its applicability to larger datasets [6–9].

In this paper, we present two techniques that speed up the training stage of the GPR algorithm. The first aims to reduce the dimensionality of the problem, and hence speed up the learning of the hyperparameters for a single covariance function. This does not change the fact that the cost of this process is $O(n^{3})$; instead it simply reduces the constant in this scaling. The second aims to enable fast Bayesian model comparison between different covariance functions while also incorporating the benefits of the first technique.

We consider maximizing the hyperlikelihood: the conditional probability of the data given a particular set of hyperparameters used to specify the covariance function.^1^ We provide an expression for the Hessian matrix of the hyperlikelihood surface and show how this can be used as a valuable tool for comparing the performance of two different covariance functions. We also present modified expressions for the hyperlikelihood, its gradient and its Hessian matrix, which have all been analytically maximized and marginalized over a single-scale hyperparameter. This analytic maximization or marginalization reduces the dimensionality of the subsequent optimization problem and hence further speeds up the training and comparison of GPs.

These techniques are useful when attempting to rapidly fit large, irregularly sampled datasets with a variety of covariance function models. The authors have previously made use of these techniques in exploring the correlation structure of the differences between complicated waveform models in the field of gravitational-wave astronomy [10,11]; this was done so that the effect of different models on the parameter inferences could be marginalized over. There, the behaviour of the data was largely unknown *a priori* and it was necessary to quantitatively compare a wide range of different covariance functions. Work in this area with larger datasets is ongoing.

In §2, we review the GPR method and discuss methods of efficiently determining a covariance function. In §2.1, we present our expression for the Hessian of the hyperlikelihood along with a discussion of how it can be used for model comparison, and in §2.2, we show how the training of the GP can be accelerated by analytically maximizing or marginalizing the hyperlikelihood over a single-scale parameter. In §3, we apply these methods to both synthetic and real datasets, and compare the computational cost to that of a full numerical evaluation of the Bayesian model evidences. Finally, a brief discussion and concluding remarks are given in §4.

## 2. Gaussian process regression and training

The technique of GPR is a method for interpolating (or extrapolating) the data contained in a *training set* D={ x, y}. The vector ***x***={*x*_*i*_ | *i*=1,2,…,*n*} is called the input vector and the output vector is given by *y*_*i*_=*f*(*x*_*i*_) for some unknown function *f*. The method works by assuming that the data have been drawn from an underlying GP $f(x)∼GP(\mu (x),k(x,x^{\prime }))$ with specified mean *μ*(*x*) (usually assumed to be zero) and covariance function *k*(*x*,*x*′).

There is freedom in specifying the covariance function; common choices, such as the squared exponential and Matérn function, include a number *m* of free hyperparameters ***θ***={*θ*_*i*_ | *i*=1,2,…,*m*} that control the properties of the GP, i.e. *k*(*x*,*x*′)=*k*(*x*,*x*′;***θ***).

The predictive power of the method comes from computing the conditional probability of the function taking a given value at some new (*n*+1)th input point x_*_, given the observed values in D and the values of the hyperparameters ***θ***. This predictive probability distribution $P(y(x_{*})\;|\;D,\theta )$ for the function at the new point is a Gaussian with mean $\bar{y}(x_{*})$ and variance [*σ*_*y*_(x_*_)]^2^ [1],

2.1 $$\bar{y}(x_{*})={\text{k}}_{*}^{T}{\text{K}}^{-1}\text{y},\;{\left[\sigma_{y}(x_{*})\right]}^{2}=k_{**}-{\text{k}}_{*}^{T}{\text{K}}^{-1}{\text{k}}_{*},$$

and

2.2 $$y(x_{*})\;|\;\{D,\theta \}∼N(\bar{y}(x_{*}),\sigma_{y}(x_{*})),$$

where we have defined the scalar, vector and matrix shorthand

2.3 $$k_{**}\equiv k(x_{*},x_{*}),\;{\left[{\text{k}}_{*}\right]}_{i}\equiv k(x_{*},x_{i})\;\mathrm{and}\;{\left[\text{K}\right]}_{ij}\equiv k(x_{i},x_{j}).$$

Since the posterior distribution for (2.2) relies upon the form of the covariance, GPR cannot be used to make definite predictions until we have fixed a method for dealing with the unknown hyperparameters ***θ***.

Ideally, we place a prior probability distribution on ***θ*** and make predictions by evaluating the integral

2.4 $$P(y(x_{*})\;|\;D)=\int d\theta P(y(x_{*})\;|\;D,\theta )P(\theta \;|\;D)=\int d\theta P(y(x_{*})\;|\;D,\theta )P(\text{y}\;|\;\text{x},\theta )P(\theta ),$$

where we have used Bayes’ theorem to obtain the second equality. We have introduced the hyperlikelihood given by

2.5 $$\ln P(\text{y}\;|\;\text{x},\theta )=-\frac{1}{2}[{\text{y}}^{T}{\text{K}}^{-1}\;\text{y}+\ln(\det\text{K})+n\ln(2\pi )],$$

which encodes the probability that the observed (training) data were drawn from a GP with covariance function *k*. The integral (2.4) is almost always analytically intractable and prohibitively expensive to evaluate numerically. A common approximate approach is to use the most probable values of the hyperparameters $\hat{\theta }$, which maximize P(θ | D) [12–14].

Assuming the prior distribution is sufficiently flat (or uninformative) over the region of interest, this is equivalent to maximizing the hyperlikelihood *P*(***y*** | ***x***,***θ***). Under this approximation, the predictive distribution becomes

2.6 $$P(y(x_{*})\;|\;D)≃P(y(x_{*})\;|\;D,\hat{\theta }),$$

which is simply the Gaussian in (2.2) with mean and variance evaluated at $\hat{\theta }$. Implementing the above procedure requires numerically maximizing the hyperlikelihood in (2.5). This can be computationally expensive; in §2.1 and §2.2, we present methods for reducing the cost of maximizing the hyperlikelihood.

### 2.1. Using the gradient and Hessian

The maximization process may be accelerated if the gradient of the hyperlikelihood is known and a gradient-based algorithm, such as a conjugate gradient method [13,15], can be used. The gradient of the logarithm of the hyperlikelihood is given by [1]

2.7 $$\partial_{\theta }\ln P(\text{y}\;|\;\text{x},\theta )=\frac{1}{2}{\text{y}}^{T}\;{\text{K}}^{-1}\cdot \partial_{\theta }\text{K}\cdot {\text{K}}^{-1}\;\text{y}-\frac{1}{2}\mathrm{Tr}({\text{K}}^{-1}\cdot \partial_{\theta }\text{K}).$$

This can be shown by differentiating (2.5) and making use of the standard results

2.8 $$\partial {\text{K}}^{-1}=-{\text{K}}^{-1}\cdot \partial \text{K}\cdot {\text{K}}^{-1},\;\partial (\det\text{K})=(\det\text{K})\mathrm{Tr}({\text{K}}^{-1}\cdot \partial \text{K}).$$

The gradient in (2.7) is useful because the rate-determining step in computing the hyperlikelihood is computing the inverse matrix **K**^−1^ (usually achieved through a Cholesky decomposition in practice), which is an $O(n^{3})$ operation. All other steps in (2.5) scale as $O(n^{2})$ or less.^2^ Once the inverse has been calculated, the gradient in (2.7) may also be evaluated in $O(n^{2})$; so in evaluating the hyperlikelihood for a large training set we can also get the gradient for negligible extra cost.

The procedure outlined above can be performed for multiple covariance functions, each yielding a different GP interpolant. It is, therefore, necessary to have a method of comparing the performance of different interpolants to decide which to use. One way to achieve this is to evaluate the (hyperprior-weighted) volume under the hyperlikelihood surface, the hyperevidence, and use this as a figure of merit for the performance. Evaluating this integral is prohibitive, so an approximation is to calculate the Hessian matrix of the ln⁡P( y |  x,θ) surface at the peak (the position and value of which have already been found) and to analytically integrate the resulting Gaussian. This procedure assumes flat (or slowly varying) hyperpriors in the vicinity of the peak, but this has already been assumed in going from (2.4) to (2.6). Differentiating the gradient in (2.7), again making use of the results in (2.8), and evaluating the derivatives at the position of peak hyperlikelihood, $\theta =\hat{\theta }$, gives the Hessian,

2.9 $$\begin{array}{rl} \partial_{\theta }\partial_{\theta^{\prime }}\ln P(\text{y}\;|\;\text{x},\theta ){|}_{\hat{\theta }} & =-\frac{1}{2}{\text{y}}^{T}[2{\text{K}}^{-1}\cdot \partial_{\theta }\text{K}\cdot {\text{K}}^{-1}\partial_{\theta^{\prime }}\text{K}\cdot {\text{K}}^{-1}-{\text{K}}^{-1}\cdot \partial_{\theta }\partial_{\theta^{\prime }}\text{K}\cdot {\text{K}}^{-1}]\text{y}\\ & \;+\frac{1}{2}\mathrm{Tr}({\text{K}}^{-1}\cdot \partial_{\theta }\text{K}\cdot {\text{K}}^{-1}\cdot \partial_{\theta^{\prime }}\text{K}-{\text{K}}^{-1}\cdot \partial_{\theta }\partial_{\theta^{\prime }}\text{K})=-\text{H}. \end{array}$$

This expression has the same advantages as the expression for the gradient; as the inverse of the covariance matrix has already been computed, the Hessian may be evaluated at negligible extra cost. The hyperlikelihood surface may therefore be approximated by the Gaussian [12,16]

2.10 $$\ln P(\text{y}\;|\;\text{x},\theta )\approx \ln P(\text{y}\;|\;\text{x},\hat{\theta })-\frac{1}{2}\Delta \theta^{T}\cdot \text{H}\cdot \Delta \theta .$$

We seek the hyperevidence, which is given by the following integral of the hyperposterior, where we have specified a prior *Π*(***θ***) on the hyperparameters;

2.11 Z(D)=∫dθΠ(θ)P(y | x,θ).

Assuming the hyperposterior is a sufficiently well-peaked distribution, with peak at position $\theta =\tilde{\theta }$, the hyperevidence may be written using the Laplace approximation [2] as

2.12 $$Z(D)\approx \Pi (\tilde{\theta })P(\text{y}\;|\;\text{x},\tilde{\theta })\sqrt{\frac{{\left(2\pi \right)}^{m}}{\det\left(\text{H}+{\text{H}}_{\Pi }\right)}}.$$

It is always possible to change the hyperparametrization so that the prior is flat in which case the hyperposterior is proportional to the hyperlikelihood.^3^ If such a hyperparametrization has been chosen then $\Pi (\tilde{\theta })=1/V$ (where *V* is the hyperprior volume, or range of integration), ***H***_*Π*_=0 and $\tilde{\theta }=\hat{\theta }$; therefore

2.13 $$Z(D)\approx \frac{P(\text{y}\;|\;\text{x},\hat{\theta })}{V}\sqrt{\frac{{\left(2\pi \right)}^{m}}{\det\text{H}}}.$$

This expression is now invariant under further changes to the hyperparameter specification which preserve the property that the prior is constant. We use hyperparametrizations with flat hyperpriors as this choice uniquely specifies the approximation in equation (2.13); although there remains the possibility that another hyperparametrization exists in which the posterior is better approximated as a Gaussian.

For two covariance functions, *k*_1_ and *k*_2_, the odds ratio may be defined as the ratio of the value of (2.13) evaluated with *k*_1_ to the value evaluated using *k*_2_, and this may be used to discriminate among competing models. The hyperprior volume *V* in (2.13) acts as an Occam factor, penalizing models with greater complexity [2]. Once suitable prior volumes have been fixed, the Hessian approximation to the hyperevidence is a computationally inexpensive means of comparing covariance functions.

The Hessian may also be used to provide error estimates for the hyperparameters; from (2.10) it can be seen that the inverse of the Hessian is the covariance matrix of the maximum hyperlikelihood estimator of the hyperparameters.

### 2.2. Partial analytic maximization

In general, covariance functions can be arbitrarily complicated, with large numbers of hyperparameters. Inevitably, simple covariance functions are the most prevalent in the literature. If there are a small number of hyperparameters, then even reducing the number of hyperparameters by one can have a great impact on the length of time taken to maximize the hyperlikelihood. In this section, we show how the hyperlikelihood for any covariance function, regardless of complexity, can be analytically maximized over an overall scale parameter, thereby reducing the number of remaining hyperparameters. We also generalize the expressions for the gradient and the Hessian found in §2.1 to this case.

Consider the following transformation of the covariance, $k({\text{ x}}_{\text{ i}},{\text{ x}}_{\text{ j}})\to \sigma_{f}^{2}k({\text{ x}}_{\text{ i}},{\text{ x}}_{\text{ j}})$; substituting this into the expression for the hyperlikelihood gives,

2.14 $$\ln P(\text{y}\;|\;\text{x},\theta )=-\frac{1}{2\sigma_{f}^{2}}{\text{y}}^{T}\;{\text{K}}^{-1}\;\text{y}-\frac{1}{2}\ln(\det\text{K})-\frac{n}{2}\ln(2\pi \sigma_{f}^{2}).$$

This function always has a unique maximum with respect to variations in $\sigma_{f}^{2}$ at the position

2.15 $${\hat{\sigma }}_{f}^{2}=\frac{1}{n}{\text{y}}^{T}\;{\text{K}}^{-1}\;\text{y};$$

at this point, the hyperlikelihood takes the value

2.16 $$\ln P_{\max}(\text{y}|\text{x},\vartheta )=-\frac{n}{2}\ln(2\pi e{\hat{\sigma }}_{f}^{2})-\frac{1}{2}\ln(\det\text{K}).$$

Equation (2.16) is to be considered as a function of the remaining *m*−1 hyperparameters ***ϑ***={***θ***∖*σ*_*f*_}. The peak evidence may now be found more easily by numerically maximizing $\ln P_{\max}$ in (2.16) with respect to the remaining parameters ***ϑ***. If a gradient-based algorithm is used, it is advantageous to have an analogous expression to (2.7) to give inexpensive derivatives. This can be found by differentiating (2.16) with respect to ***ϑ***, making use of the results in (2.8),

2.17 $$\partial_{\vartheta }\ln P_{\max}(\text{y}|\text{x},\vartheta )=\frac{1}{2{\hat{\sigma }}_{f}^{2}}{\text{y}}^{T}\;{\text{K}}^{-1}\cdot \partial_{\vartheta }\text{K}\cdot {\text{K}}^{-1}\;\text{y}-\frac{1}{2}\mathrm{Tr}({\text{K}}^{-1}\cdot \partial_{\vartheta }\text{K}).$$

These are not the same as the derivatives in (2.7).

As well as maximizing, we can also consider marginalizing over *σ*_*f*_ [16]. As we are marginalizing over a scale parameter we use the (improper) Jeffreys prior *P*(*σ*_*f*_)=*c*/*σ*_*f*_ [17]. The result is equal to the maximized form, up to a multiplicative constant,

2.18 $$P_{\mathrm{marg}}(\text{y}\;|\;\text{x},\theta )=\int_{0}^{\infty }d\sigma_{f}\frac{c}{\sigma_{f}}P(\text{y}\;|\;\text{x},\theta )=\frac{c}{2}{\left(\frac{2e}{n}\right)}^{n/2}\Gamma \left(\frac{n}{2}\right)P_{\max}(\text{y}|\text{x},\theta ).$$

As before, once the peak hyperlikelihood has been found, the Hessian at the peak position can aid in model comparison. In this case, the Hessian should be calculated using the second derivatives of $\ln P_{\mathrm{marg}}$. However, we may instead differentiate $\ln P_{\max}$, as this differs only by a constant which will cancel when using the Hessian to compare two models. Differentiating (2.17) with respect to ***ϑ***′,^4^

2.19 $$\begin{array}{rl} \partial_{\vartheta }\partial_{\vartheta^{\prime }}\ln P_{\mathrm{marg}}{|}_{\hat{\vartheta }} & \propto \frac{1}{2n{\hat{\sigma }}_{f}^{4}}{\text{y}}^{T}\;{\text{K}}^{-1}\cdot \partial_{\vartheta }\text{K}\cdot {\text{K}}^{-1}\;\text{y}\times {\text{y}}^{T}{\text{K}}^{-1}\cdot \partial_{\vartheta^{\prime }}\text{K}\cdot {\text{K}}^{-1}\;\text{y}\\ & \;-\frac{1}{2{\hat{\sigma }}_{f}^{2}}{\text{y}}^{T}[2{\text{K}}^{-1}\cdot \partial_{\vartheta }\text{K}\cdot {\text{K}}^{-1}\cdot \partial_{\vartheta^{\prime }}\text{K}\cdot {\text{K}}^{-1}-{\text{K}}^{-1}\cdot \partial_{\vartheta }\partial_{\vartheta^{\prime }}\text{K}\cdot {\text{K}}^{-1}]\text{y}\\ & \;+\frac{1}{2}\mathrm{Tr}({\text{K}}^{-1}\cdot \partial_{\vartheta }\text{K}\cdot {\text{K}}^{-1}\cdot \partial_{\vartheta^{\prime }}\text{K}-{\text{K}}^{-1}\cdot \partial_{\vartheta }\partial_{\vartheta^{\prime }}\text{K}). \end{array}$$

Again, these are not the same as the derivatives in (2.9). These expressions for the gradient and the Hessian of the hyperlikelihood, maximized or marginalized over $\sigma_{f}^{2}$, share the same advantages as the analogous expressions in §2: they may be evaluated in $O(n^{2})$ time once the hyperlikelihood itself has been evaluated in $O(n^{3})$ time.

## 3. Numerical results

In order to perform model comparison calculations between competing covariance functions, we must first specify at least two different covariance functions. We choose the two functions in (3.1) and (3.2), where (*t*,*t*′)≡(*x*,*x*′). These functions are both based on the periodic covariance function proposed by [2]. The first function *k*_1_ is the product of a single periodic component with time scale *T*_1_ and a simple compact-support polynomial covariance function [18] to describe any non-periodic component of the data. The choice of a compact-support covariance function is especially useful when working with large datasets; this is precisely the situation where the techniques described above are also designed to be of maximum benefit. The second function *k*_2_ includes an additional periodic component with time scale *T*_2_. In order to avoid double-counting in *k*_2_, we impose the constraint *T*_2_≥*T*_1_. Both covariance functions also include an uncorrelated noise term; we define this in such a way that *σ*_*f*_ remains an overall scale hyperparameter which can be maximized or marginalized over analytically as described in §3.2.

3.1 $$\begin{array}{rl} k_{1}(t,t^{\prime }) & =\sigma_{f}^{2}C\left(\frac{|t-t^{\prime }|}{T_{0}}\right)\exp\left[-\frac{2}{l_{1}^{2}}\sin^{2}\left(\frac{\pi (t-t^{\prime })}{T_{1}}\right)\right]+\sigma_{f}^{2}\sigma_{n}^{2}\delta_{tt^{\prime }}, \end{array}$$

3.2 $$\begin{array}{rl} k_{2}(t,t^{\prime }) & =\sigma_{f}^{2}C\left(\frac{|t-t^{\prime }|}{T_{0}}\right)\exp\left[-\frac{2}{l_{1}^{2}}\sin^{2}\left(\frac{\pi (t-t^{\prime })}{T_{1}}\right)-\frac{2}{l_{2}^{2}}\sin^{2}\left(\frac{\pi (t-t^{\prime })}{T_{2}}\right)\right]+\sigma_{f}^{2}\sigma_{n}^{2}\delta_{tt^{\prime }} \end{array}$$

3.3 $$\begin{array}{rl} \;\text{and}\;C(\tau ) & =\left\{ \begin{array}{ll} {\left(1-\tau \right)}^{5}\frac{48\tau^{2}+15\tau +3}{3} & \tau <1\\ 0 & \tau >1 \end{array}\right.. \end{array}$$

The covariance functions are completely specified by the hyperparameters *σ*_*f*_ (overall scale), *T*_*j*_ (*j*=0,1,2; time scales) and *l*_*j*_ (*j*=1,2; smoothing parameters for the periodic components). The noise parameter *σ*_*n*_ could also be taken to be a hyperparameter; instead, for simplicity, we here take *σ*_*n*_ to be fixed. As *σ*_*n*_ appears in *k* multiplied by the overall scale, *σ*_*f*_, fixing *σ*_*n*_ is roughly equivalent to specifying a fixed fractional error.

We want to perform model comparison using the Laplace approximation outlined previously. This technique requires reparametrizing the covariance function such that the hyperpriors are flat. For the time-scale hyperparameters, which are dimensionful, we choose to use the scale-invariant Jeffreys prior, *P*(*T*_*j*_)∝1/*T*_*j*_. This prior is improper if the range of *T*_*j*_ is (0,∞), so we restrict the range to (*δt*,*ΔT*), where *δt* and *ΔT* are respectively the smallest and largest separations between the sampling points. If there was a time scale in the problem outside of this range, we would be unable to resolve it from the data. We now seek a transformation *ϕ*_*j*_≡*ϕ*_*j*_(*T*_*j*_) to a new hyperparameter *ϕ*_*j*_ such that the prior is flat in this parameter, *P*(*ϕ*_*j*_)=const. The conservation of probability gives a differential equation relating the two

3.4 $$P(T_{j})\;dT_{j}=P(\phi_{j})\;d\phi_{j}\;\Rightarrow \;T_{j}=\exp\left(\frac{\phi_{j}}{A_{j}}\right),\;\{j=0,1,2\},$$

where the *A*_*j*_s are constants which we can set equal to 1. The range of these new hyperparameters is $\phi_{j}\in (\ln(\delta t),\ln(\Delta T))$ and $P(\phi_{j})=1/\ln(\Delta T/\delta t)$.

For the smoothness parameters *l*_*j*_, we choose to use lognormal priors, $P(l_{j})=\exp[-{\left(\mu -\log l_{j}\right)}^{2}/(2\sigma_{l}^{2})]/\sqrt{2\pi \sigma_{l}^{2}}$, with mean *μ*=1 and variance $\sigma_{l}^{2}=4$. As before, we seek a transformation to some new hyperparameters *ξ*_*j*_ in which the prior is flat. The desired transformation is given by

3.5 $$l_{j}=\exp[\mu +\sqrt{2}\sigma_{l}{\mathrm{erf}}^{-1}(2\xi_{j})],\;\{j=1,2\},$$

where *ξ*_*j*_∈(−0.5,0.5).

### 3.1. Synthetic data

**Figure 1.**
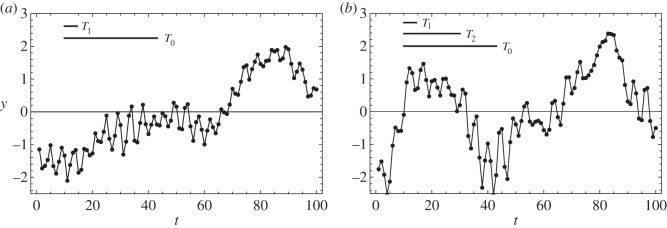
Realizations of the GPs *k*_1_(*t*,*t*′) and *k*_2_(*t*,*t*′) from (3.1) and (3.2) for values of *t*=1,2,3…,100 are shown in the (*a*,*b*) panels, respectively. The horizontal black lines indicate the length scales associated with the different terms in the covariance functions. The hyperparameters for *k*_1_ were chosen to be *σ*_*f*_=1, *ϕ*_0_=3.5, *ϕ*_1_=1.5 and *x*_1_=0. The hyperparameters for *k*_2_ were chosen to be the same as for *k*_1_ and *ϕ*_2_=3 and *x*_2_=0. In both cases, the noise was fixed to *σ*_*n*_=10^−2^.

Shown in figure 1 are realizations of GPs with covariance functions *k*_1_ and *k*_2_.^5^ In order to perform test model comparison calculations, a realization of the *k*_2_ GP with *n* points was drawn and analysed using both the *k*_1_ and *k*_2_ covariance functions. For each covariance, the peak hyperlikelihood was found by numerically maximizing (2.14) using a conjugate gradient method, making use of the gradient in (2.17). The hyperevidence was estimated using (2.13) and the expression for the Hessian in (2.19); the results are summarized in table 1. To verify the accuracy of this estimate, the hyperevidence was also integrated numerically using MultiNest [19–21], which implements a nested sampling algorithm [22]. This was repeated for three different values of *n* (in the case *n*=100, the synthetic data are plotted in the right-hand panel of figure 1), and the results are also summarized in table 1.

**Table 1.** A summary of the results of the analysis of synthetic data for three different-sized datasets. The first set of two columns is for a dataset drawn from the *k*_2_ covariance function and analysed with the *k*_1_ covariance function. The first column is the estimated hyperevidence using the Laplace approximation where Z is as given in equation (2.13), while the second is the numerically calculated hyperevidence. The second set of two columns shows results for the same data, but analysed with the *k*_2_ covariance function. The final pair of columns shows the log Bayes factor, $\ln B\equiv \ln Z^{k_{2}}-\ln Z^{k_{1}}$, calculated using the approximate and numerical values for the hyperevidence.

*n*	$\ln Z_{\mathrm{est}}^{k_{1}}$	$\ln Z_{\mathrm{num}}^{k_{1}}$	$\ln Z_{\mathrm{est}}^{k_{2}}$	$\ln Z_{\mathrm{num}}^{k_{2}}$	$\ln B_{\mathrm{est}}$	$\ln B_{\mathrm{num}}$
30	−17.77	−17.87±0.08	−*18.82*	−17.73±0.09	−1.05	0.14±0.12
100	−20.17	−20.17±0.10	−19.22	−19.22±0.11	0.95	0.95±0.15
300	−49.94	−50.12±0.11	−40.21	−40.36±0.13	9.73	9.76±0.17

**Figure 2.**
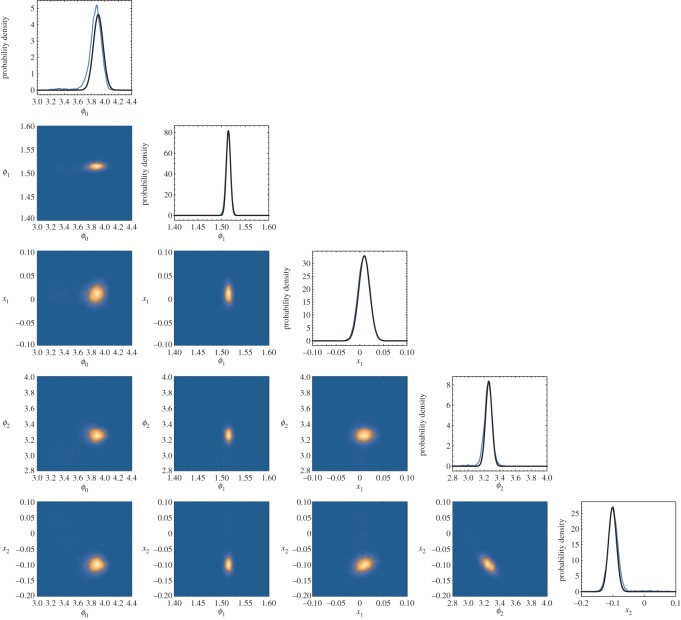
The one- and two-dimensional marginalized posterior distributions on the hyperparameters of the *k*_2_ covariance function obtained from the largest (*n*=300) synthetic dataset. The posterior is well approximated as a normal distribution. Shown in the black curves in the one-dimensional marginalized posterior distributions along the diagonal are the normal approximations obtained by using the techniques described in §2 to maximize and find the Hessian. Using the Hessian to approximate the integral of this distribution (the hyperevidence) leads to an error of approximately 10% (table 1).

From table 1 it can be seen that as *n* is increased, the Bayes factors increasingly favour the more complicated covariance function (and in this case the correct covariance function from which the data was drawn). In almost all cases, the Laplace approximation gives a value $\ln Z_{\mathrm{est}}$ which is in agreement at better than 2*σ* with the numerically integrated value $\ln Z_{\mathrm{num}}$. There is one exception which is highlighted in italic; this occurs for the most complicated covariance function (with the largest number of hyperparameters) and when the number of data points is smallest. In this situation, it would be expected that the posterior distribution on the hyperparameters may be highly multimodal and/or exhibit strong degeneracies (both of these expectations were confirmed by examining the posterior distribution on the hyperparameters returned by MultiNest). This exceptional case serves to highlight situations in which the Laplace approximation should not be trusted. The MultiNest posteriors in all other cases were verified to be well approximated by a single Gaussian mode. Figure 2 shows the posterior distribution for the parameters of *k*_2_ obtained from the largest (*n*=300) synthetic dataset.

Our method of model comparison is proposed as a faster alternative to model comparison using numerically evaluated Bayes factors. Simply comparing the peak hyperlikelihood (marginal likelihood) values would also give a measure of the goodness of fit, but this tends to favour more complex models and incurs the risk of overfitting. More sophisticated methods of model selection exist in the literature (see [23,24] and references within), e.g. the comparison of models based on estimated predictive criteria [25–27], or the construction of a larger reference model and the subsequent selection of a simpler submodel with similar predictions [28,29]. A detailed numerical comparison to these methods is left for future investigation.

The $\ln Z_{\mathrm{num}}$ values in table 1, evaluated using MultiNest, required between 20 000 and 50 000 likelihood evaluations. The maximization routines typically took fewer than 100 likelihood evaluations to find the peak, and then one additional evaluation to calculate the Hessian and hence $\ln Z_{\mathrm{est}}$. In order to guard against the possibility of the maximization routines becoming trapped in local maxima, as opposed to the global maximum, the algorithm was run multiple times from randomly selected starting positions. The typical number of runs required to find the global maximum was approximately 10. After these duplicate runs are accounted for, the speed-up factor in calculating $\ln Z_{\mathrm{est}}$ compared with $\ln Z_{\mathrm{num}}$ was between 20 and 50 in all cases.

### 3.2. Tidal data from Woods Hole

**Figure 3.**
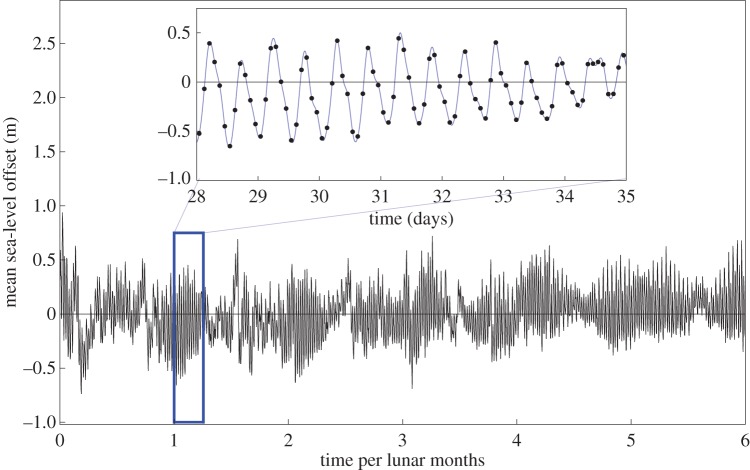
Shown in the main figure are six lunar months of tidal height data (black), from which the lunar tidal cycle can be discerned. Shown in the inset plot are several days of the tidal data (black points), from which the daily cycles can be clearly seen. Overlaid in the inset plot are both GP interpolants (blue), which are identical on this time scale.

In order to illustrate the effectiveness of the techniques described above on real data, we consider several tidal datasets of different sizes from Woods Hole, MA, USA [30].^6^ We consider the mean sea-level offset recorded between 3 January and 15 June 2014, six lunar months, sampled at 2 h intervals (giving *n*=1968 data points). This is plotted in figure 3. We also consider a smaller subset of the data (the first lunar month), with *n*=328 data points.^7^

We interpolate the data using the two covariance functions in (3.1) and (3.2); these functions are well suited to the data, as we expect the sea level to contain harmonics of the various time scales associated with the daily, monthly and yearly cycles of the tides. For simplicity, we fix *σ*_*n*_=10^−2^, which is the typical fractional error in the sea-level measurements. As in §3.1, we reparametrize the covariance functions so that the *T*_*j*_ have Jeffreys priors, and the smoothness parameters have lognormal priors. We use a conjugate gradient maximization algorithm with (2.17) and the Hessian in (2.19) to evaluate the volume in (2.13) and perform model comparison between the two covariance functions.

For the smaller dataset, we find the time scale *T*_1_=(12.8±0.2) h with *k*_1_, which corresponds to the two main tides per day. With *k*_2_, we find the time scales *T*_1_=(12.44±0.07) h and *T*_2_=(24.3±1.0) h; the second time scale corresponds to the height difference between the first and second tides of the day. The two time-scale model is highly favoured with a log Bayes factor of 57.8.

For the larger dataset, we find *T*_1_=(12.80±0.11) h with *k*_1_, and *T*_1_=(12.40±0.03) h and *T*_2_=(23.3±0.3) h with *k*_2_. In all cases, the (squared) errors are estimated using the diagonal components of the inverse Hessian; it can be seen that the time scales are more precisely measured for the larger dataset, as expected. The two time-scale model is even more conclusively favoured for the larger dataset, with a log Bayes factor of 538. We also find a number of subsidiary hyperlikelihood peaks associated with other time scales in the data, but all subsidiary peaks are strongly suppressed relative to the global peak (by at least Δln⁡P of approx. 100) and so we expect our Bayes factor estimates to be robust.

Sea-level data are known to contain a large number of different frequencies, which necessitates the use of harmonic analysis in tidal modelling; the number of constituents included in tide prediction calculations has increased from tens [31] to thousands [32] over the past century. Clearly any *k*_2_-like covariance function with fewer than 10 time scales is simplistic, but the construction of a more detailed tidal model is beyond the scope of this paper.^8^

The number of evaluations of (2.13) needed to obtain these results was comparable with the numbers for the synthetic data discussed in §3.1. However, each evaluation here was more expensive (approx. 10 s) due to the size of the dataset. Based on the speed-ups found in §3.1, it would be expected that multinest would take up to approximately one week to calculate the Bayes factor.

Shown in the inset plot in figure 3 are the two interpolants from *k*_1_ and *k*_2_ for the larger dataset, which both perform equally well on the time scale of one week. These interpolants, which are the result of the regression analysis, may be used to estimate the tidal height at a time where a measurement is not available.

## 4. Summary

We have described some simple ways in which the computationally expensive training stage of implementing GPR can be accelerated. The analytic maximization of the hyperlikelihood over a single-scale hyperparameter of the covariance function aids in speeding up the maximization of the hyperlikelihood by reducing the dimensionality of the problem; the advantages of this will be most keenly felt in (common) problems where relatively simple covariance functions are used. Meanwhile, the analytic evaluation of the Hessian matrix, either in the manner of (2.9) or (2.19), aids in speeding up the process of model comparison between different types of covariance function. We have successfully demonstrated these techniques on a synthetic dataset where the data was drawn from one of two covariance functions under consideration. In the case of the synthetic data, the size of the dataset was limited to fewer than 300 points, so that the results could be verified by using the MultiNest algorithm to numerically sample and integrate the posteriors. We also demonstrated the techniques by applying them to a larger real dataset of mean sea-level measurements, where the full MultiNest calculation would have taken too long to perform. It is to be hoped that these techniques will aid in the wider application of GP methods to larger datasets.

## Footnotes

1 This is often referred to as the marginal likelihood. In order to avoid confusion with distributions over the model parameters, we prefer to consistently use the ‘hyper’ prefix to denote probability distributions connected to the inference of the hyperparameters.

2 As described in a footnote in [1], the matrix–matrix products in (2.7) should not be evaluated directly, as this is an $O(n^{3})$ operation. Rather, the first term should be evaluated in terms of matrix–vector products, and, in the second term, only the diagonal elements that contribute to the trace need to be calculated; these are both $O(n^{2})$ operations.

3 For example, if the original prior on the parameters ***θ*** is *p*(***θ***), we can define $\theta_{i}^{\prime }(\theta )=\int_{-\infty }^{\theta_{i}}p(\theta_{i}\;|\;\theta_{i+1},\ldots ,\theta_{m})\;d\theta_{i}$ and then *p*(***θ*′**) is a constant.

4 Here, ${\hat{\sigma }}_{f}$ retains its maximum ln⁡P value from (2.15), although the new maximum $\ln P_{\mathrm{marg}}$ value has actually now shifted to ${\left({\hat{\sigma }}_{f}^{\prime }\right)}^{2}=n{\hat{\sigma }}_{f}^{2}/(n-1)$ due to the effect of the hyperprior. For large datasets (*n*≫1), the difference between the two is negligible (the hyperprior becomes uninformative as it is overwhelmed by the hyperlikelihood).

5 The code, optimized for use on a GPU, and the synthetic used to produce these numerical results is available at http://hdl.handle.net/10283/1924.

6 Data from the National Oceanic and Atmospheric Administration, http://tidesandcurrents.noaa.gov/waterlevels.html (accessed August 2015).

7 This dataset is regularly sampled in time, and therefore, the covariance matrix will be a Toeplitz matrix. This structure could be exploited to accelerate the inversion of the covariance matrix; we choose not to use this here so that our code can be applied to irregularly sampled data in arbitrary dimensions.

8 We have conducted preliminary investigations of a three time-scale model. The hyperlikelihood surface for this covariance function is more structured and non-Gaussian than for *k*_2_ and *k*_1_. Estimates of the Bayes factors indicate that the inclusion of additional time scales is favoured, as expected based on the known large number of modes present.

## Data accessibility

The original source of the tidal data is the National Oceanic and Atmospheric Administration (http://tidesandcurrents.noaa.gov/waterlevels.html, accessed August 2015). The subset of data used here, as well as our synthetic datasets and our code is available at http://hdl.handle.net/10283/1924.
